# Septoplasty and Nasal Packs From Otolaryngologists’ Perspectives: A Cross-Sectional Survey

**DOI:** 10.7759/cureus.54691

**Published:** 2024-02-22

**Authors:** Balqees Alghanem, Nawaf Almotairi, Ahmad Alrasheedi

**Affiliations:** 1 Otolaryngology - Head and Neck Surgery, Kuwait Institute for Medical Specializations, Kuwait, KWT; 2 Otorhinolaryngology - Head and Neck Surgery, Farwaniya Hospital, Kuwait, KWT

**Keywords:** nasal packs, silastic splints, trans-septal suturing, septoplasty, otolaryngology

## Abstract

Introduction

Septoplasty is a commonly performed surgery by otolaryngologists. This study examines the routine use of non-absorbable nasal packs in the postoperative period in Kuwait. Our aim is to assess the views of otolaryngologists in Kuwait regarding the routine use of non-absorbable nasal packing after septoplasty with or without turbinoplasty.

Materials and methods

A cross-sectional study, an anonymous survey, consisting of 10 questions, using Google Forms was conducted. It was distributed via WhatsApp and in person hard copies to otolaryngologists - head and neck surgeons in Kuwait. Data were securely stored and analyzed using Stata (StataCorp LLC, College Station, Texas) software.

Results

Fifty participants have completed the survey. The majority were routinely performing septoplasty for a symptomatic deviated nasal septum (n= 47, 94%). Out of those, 43% (n = 21) usually use silastic splints to prevent postoperative complications, including bleeding, hematoma formation, and adhesions. The use of quilting sutures for the same purpose was done by 21% (n = 10), and 17% (n = 8) used non-absorbable nasal packs. Only two participants believed that no specific intervention was required. More than half used postoperative nasal packs in the case of intra-operative bleeding (n=26, 52%), while 18% (n = 9) believed that it is only necessary in selected cases with predisposing factors to bleeding. Ninety-two percent (n = 46) thought that pain and discomfort were associated with the use of non-absorbable nasal packs, and 50% (n = 25) believed that it could cause hemodynamic instability. Moreover, 66% (n = 33) agreed that quilting or trans-septal sutures can safely substitute nasal packs following septoplasty. Awareness of recent updates on the complications related to nasal packing varied, with more than half of the otolaryngologists being knowledgeable (n = 29, 58%).

Discussion

Septoplasty, a common surgical intervention for correcting a misaligned nasal septum, addresses various symptoms, such as nasal obstruction, obstructive sleep apnea, epistaxis, and headache. While the practice of nasal packing for 24-48 hours post-septoplasty is widespread, it remains a topic of controversy. Current literature supports the continued use of nasal packing for patients at high risk of bleeding. However, potential drawbacks, including increased pain, headache, and prolonged hospital stay, raise concerns about its overall benefits. An alternative approach, utilizing quilting sutures without nasal packs, has shown promising results in comparative studies, demonstrating less pain and minimal bleeding. Additionally, trans-septal sutures emerge as a safe alternative, minimizing dead space and reducing post-operative complications.

Conclusion

It appears that otolaryngologists in Kuwait have diverse opinions with regard to nasal packing following septoplasty. Further research is needed to establish evidence-based guidelines for this common procedure.

## Introduction

Septoplasty is a commonly performed surgical procedure in otolaryngology, addressing the correction of a misaligned nasal septum and alleviating various associated symptoms, such as nasal blockage, obstructive sleep apnea, epistaxis, and headache [1]. Despite its widespread use, the routine application of non-absorbable nasal packing post-operatively has become a subject of ongoing debate among otolaryngologists.

This study aims to assess otolaryngologists' perspectives on the necessity of non-absorbable nasal packing after septoplasty, with or without turbinoplasty. By investigating the thoughts and viewpoints of medical experts in this field, we aim to illuminate preferred post-operative practices. Our goal is to contribute to the ongoing discourse on optimal management, specifically focusing on whether to use non-absorbable nasal packs or alternative methods for patients undergoing septoplasty.

## Materials and methods

Study design

This research employed a cross-sectional study design to comprehensively explore otolaryngologists' perspectives on the use of non-absorbable nasal packing after septoplasty. The study utilized an anonymous survey comprising 10 questions to capture a diverse range of opinions and practices among participants (Table 1).

**Table 1:** Survey Questions on Clinical Practices in Septoplasty Regarding the Use of Septoplasty and Alternative Methods.

Survey
1. Do you consent to participate in this questionnaire?
Yes, No
2. What is your job title?
Assistant registrar, Registrar, Senior registrar, Specialist, Senior specialist, Consultant
3. Do you usually perform septoplasty for symptomatic deviated nasal septum?
Yes, No
4. What is your preferred method to decrease the risk of post-septoplasty complications (bleeding, hematoma, adhesions)? You can choose more than one answer.
None, Quilting or trans-septal sutures, Silastic splints for one to two weeks, Nasal packs for 24 to 48 hours
5. What is the importance of post-septoplasty nasal packing?
It is done routinely, In selective cases (HTN or Bleeding tendencies) to minimize bleeding risk, Depends on the intra-operative bleeding, Only if turbinoplasty/partial turbinectomy is performed, If others, please specify (-------------------------------)
6. In your opinion, does nasal packing decrease the risk of post-septoplasty bleeding?
Yes, No
7. Is post-operative nasal packing associated with a higher risk of pain and discomfort when compared to no packing method?
Yes, No
8. Do you think that a nasal pack can cause hemodynamic instability in some patients? (tachycardia or increase in blood pressure)
Yes, No
9. Do you think that trans-septal or quilting sutures can be safely used as an alternative to routine nasal packing after septoplasty?
Yes, No
10. Did you check the recent literature that discussed the complications of post-septoplasty nasal packs and the available alternatives?
Yes, No

Survey development and distribution

The questionnaire was carefully crafted to address key aspects of post-septoplasty practices. Google Forms was selected as the platform for survey administration due to its user-friendly interface and efficient data collection capabilities. The survey link was distributed via WhatsApp, a widely used communication platform among healthcare professionals. Additionally, hard copies of the questionnaire were distributed in person to otolaryngologists - head and neck surgeons in Kuwait to ensure inclusivity and facilitate participation.

Participant recruitment

Sixty otolaryngologists in Kuwait were approached to partake in the study, ensuring a diverse representation of professionals in the field. This sample size was considered adequate for a comprehensive understanding of the perspectives within the local medical community. Out of the 60 approached, 50 respondents actively participated in the study, providing valuable insights into the post-septoplasty practices and opinions among the surveyed otolaryngologists.

Data collection period

Data collection transpired between October and December 2023. This timeframe was selected to capture a representative snapshot of current practices and opinions among otolaryngologists in Kuwait.

Data storage and security

Survey data were safely stored on OneDrive, a secure cloud platform with strong data protection features. We prioritized confidentiality and anonymity to promote honest participant responses.

Data analysis

Data analysis was conducted using State software (StataCorp LLC, College Station, Texas) [2], employing statistical methods to derive meaningful insights from the collected responses. Frequencies and percentages were utilized during the data analysis process. This analytical approach aimed to identify patterns, trends, and variations in otolaryngologists' perspectives on the use of non-absorbable nasal packing after septoplasty.

Content validity confirmation

To ensure the relevance and effectiveness of the survey questions, content validity was established through the opinions of two experts. The questionnaire underwent review by two experts in the field of otolaryngology, and their feedback was incorporated to enhance the survey's effectiveness in capturing detailed insights.

## Results

**Figure 1:**
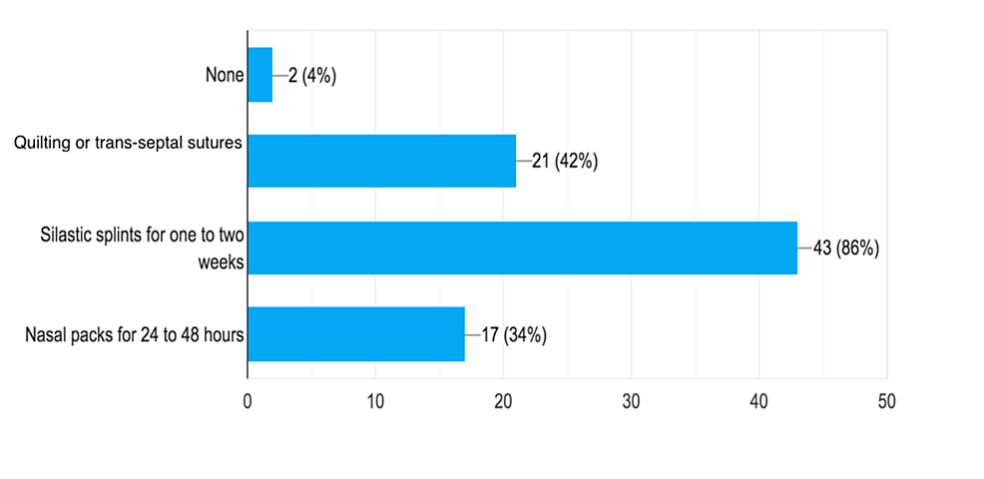
Preferred Methods for Minimizing Complications After Septoplasty.

Fifty otolaryngologists participated in the survey, providing their opinions on non-absorbable nasal packing after septoplasty, with or without turbinoplasty. The majority routinely performed septoplasty for a symptomatic deviated nasal septum (n = 47, 94%). Among the surveyed otolaryngologists, 43 (86%) believed that using silastic splints for one to two weeks was an effective approach to reduce the risk of post-operative complications, including bleeding, hematoma, and adhesions. However, 21 (42%) surgeons suggested quilting or trans-septal sutures as an alternative. Seventeen (34%) considered non-absorbable nasal packs for 24-48 hours an effective approach to minimize post-septoplasty complications, while only two expressed the view that no specific intervention was needed (Figure 1).

Fifty-two percent (n = 26) of the surveyed otolaryngologists indicated that they consider the importance of using nasal packs to depend on the amount of intraoperative bleeding, while 18% (n = 9) believed that it is only necessary in selected cases with a predisposition to postoperative bleeding, including bleeding tendency and hypertension. Twelve percent (n = 6) reported that nasal packing was routinely performed, and 10% (n = 5) mentioned its use for other reasons, such as hemostasis, healing, or epistaxis.

**Figure 2:**
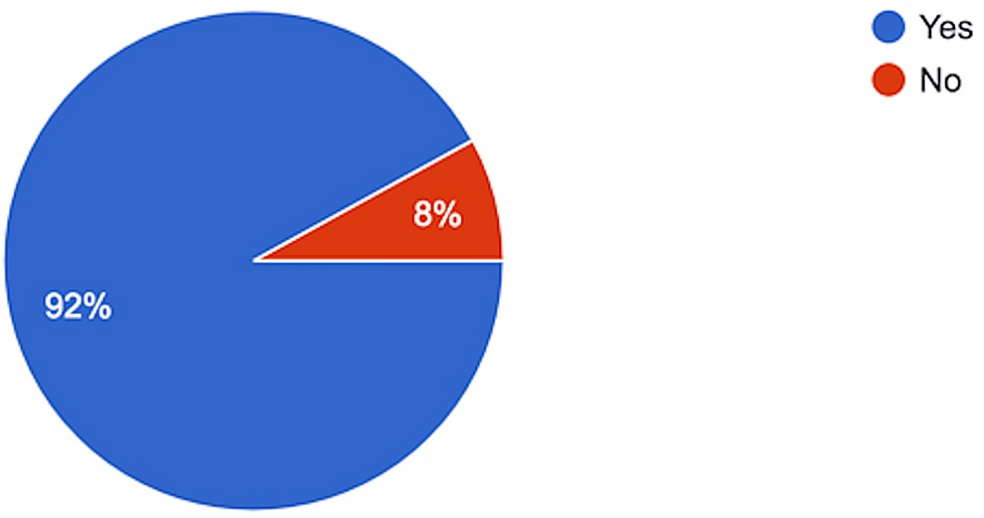
Answers to the Question: Is Post-operative Nasal Packing Associated With a Higher Level of Pain & Discomfort When Compared to the Non-packing Method?

Twenty-six otolaryngologists (52%) believed that nasal packing reduces the risk of post-septoplasty bleeding, while 24 48%) disagreed. Regarding pain and discomfort, 92% (n = 46) stated that post-operative nasal packing was linked to higher levels of pain and discomfort among patients (Figure 2).

**Figure 3:**
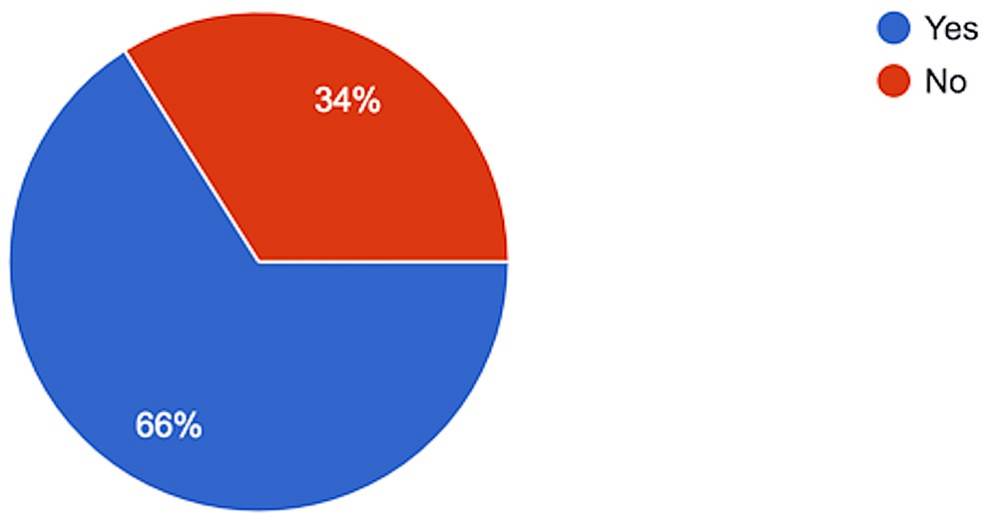
Answers to the Question: Do You Think That Transeptal or Quilting Suture Can Be Safely Used as an Alternative to Routine Nasal Packs After Septoplasty?

Half of the respondents (n = 25, 50%) believed that post-operative nasal packing can lead to features of hemodynamic instability, including tachycardia or hypertension in some patients. Sixty-six percent (n = 33) believe that trans-septal or quilting sutures could safely replace routine nasal packing after septoplasty (Figure 3).

Within the surveyed group, the knowledge of the recent literature discussing complications arising from post-septoplasty nasal packs and potential alternatives varied; 58% (n = 29) were knowledgeable, whereas 42% (n = 21) were not.

## Discussion

Septoplasty is the surgical correction of misaligned nasal septum. It is a common surgical intervention performed in otolaryngology departments [1]. Septoplasty is mainly performed to correct septal deformities that lead to nasal obstruction, obstructive sleep apnea, epistaxis, and headache. Several surgical techniques can be used, including endoscopic surgery and endonasal or open approaches. The surgical steps involve hydro-dissection by infiltrating local anesthesia on both sides of the septum, followed by a Killian or hemi-transfixion incision. Mucoperichondrial and mucoperiosteal flaps are elevated with subsequent removal of the deviated septal components, preserving an “L-strut” shape of the quadrangular cartilage. The incision is closed with absorbable sutures and bilateral silastic splints are placed endonasally. Patients are usually discharged on the same day and followed up after one to two weeks for the removal of silastic splints [3]. Patients with septal deformity report an improvement in their quality of life and a reduced need for medications after septoplasty [4,5].

Nasal packing for 24-48 hours after septoplasty is a common practice that remains controversial. In our study, 14% were routinely using nasal packs in the post-operative period. This practice is thought to decrease the rates of post-operative bleeding and hematoma formation, but the risk of bleeding after a septoplasty without nasal packing is low [6]. Current literature suggests that nasal packing after septoplasty should be continued for patients with a higher risk of bleeding because it does not affect the outcome [7]. However, the harm caused by post-operative packing may be greater than its benefits. Patients who underwent nasal packing after septoplasty reported high pain levels, headaches, and prolonged hospital stay [8,9]. In a study that involved 50 patients who underwent septoplasty. In half of them, nasal packs were not used, and quilting suture was utilized. This group reported experiencing less pain and minimal bleeding compared to the other half of the patients who had nasal packs inserted [7]. Additional complications of nasal packing are that it may affect the hemodynamic state of the patient as it can increase the heart rate, blood pressure, and nocturnal blood pressure in previously healthy patients [10].

Trans-septal sutures can be safely used as an alternative to nasal packing after septoplasty [11]. Trans-septal or quilting sutures minimize the dead space created by surgery and decrease the risk of post-operative pain, headache, sleep disturbance, and epiphora that result from routine nasal packing without changing the outcome [12-14].

Among the surveyed group in our study, 66% thought that quilting sutures could be used safely as an alternative to routine nasal packs. Recent literature proved that the use of quilting sutures without packs, clips, or splints decreases the risk of septal hematoma, synechia, and post-operative bleeding [15].

Limitations

This cross-sectional observational study offers valuable insights into the perspectives of otolaryngologists regarding nasal packing after septoplasty. However, it is important to consider the specific context of Kuwait. Given the limited number of otolaryngologists practicing in Kuwait, a sample size of 50 participants can be considered representative of the local medical community. Given Kuwait's status as a relatively small country, this sample size aligns with the unique characteristics of the study population and may adequately reflect perspectives within this specific context. It is crucial to acknowledge that the generalizability of the findings beyond Kuwait may therefore be limited. Additionally, the survey methodology employed, including distribution via WhatsApp and in-person handouts, might have introduced selection bias, potentially excluding voices that do not participate in these channels. Moreover, this study relied on self-reported data from medical professionals and lacked objective clinical measurements. Patient perspectives on the impact of nasal packing are lacking, and a more comprehensive exploration of complications related to nasal packing is warranted.

## Conclusions

Our study highlights that most otolaryngologists recommend nasal packing only for patients with certain medical conditions, such as bleeding disorders or hypertension. The routine use of nasal packing after septoplasty may not always be necessary and may cause pain and discomfort. Instead, trans-septal sutures could be a better option for reducing complications while maintaining surgical success. With advances in otolaryngology, tailoring postoperative care for individual patients is crucial for comfort and fewer complications. Future research should assess patient-reported outcomes, including satisfaction, comfort, and quality of life following different postoperative techniques, which would provide valuable insights into optimizing patient care and enhancing overall outcomes in septoplasty procedures.
